# Pharmacogenomic profiling reveals molecular features of chemotherapy resistance in IDH wild-type primary glioblastoma

**DOI:** 10.1186/s13073-023-01165-8

**Published:** 2023-03-13

**Authors:** Yoonhee Nam, Harim Koo, Yingxi Yang, Sang Shin, Zhihan Zhu, Donggeon Kim, Hee Jin Cho, Quanhua Mu, Seung Won Choi, Jason K. Sa, Yun Jee Seo, Yejin Kim, Kyoungmin Lee, Jeong-Woo Oh, Yong-Jun Kwon, Woong-Yang Park, Doo-Sik Kong, Ho Jun Seol, Jung-Il Lee, Chul-Kee Park, Hye Won Lee, Yeup Yoon, Jiguang Wang

**Affiliations:** 1 Division of Life Science and State Key Laboratory of Molecular Neuroscience, The Hong Kong University of Science and Technology, Clear Water Bay, Kowloon, Hong Kong SAR, China; 2 Institute for Refractory Cancer Research, Research Institute for Future Medicine, Samsung Medical Center, Seoul, South Korea; 3 Department of Biomedical Sciences, Korea University College of Medicine, Seoul, South Korea; 4 Department of Cancer Biomedical Science, Graduate School of Cancer Science and Policy, National Cancer Center, Goyang, South Korea; 5 Department of Clinical Research, Research Institute and Hospital, National Cancer Center, Goyang, South Korea; 6 Department of Chemical and Biological Engineering, The Hong Kong University of Science and Technology, Clear Water Bay, Kowloon, Hong Kong SAR, China; 7 Department of Biomedical Convergence Science and Technology, School of Convergence, Kyungpook National University, Daegu, South Korea; 8 Cell and Matrix Research Institute, Kyungpook National University, Daegu, South Korea; 9 Program for Mathematical Genomics, Department of Systems Biology, Columbia University, New York, NY USA; 10 Department of Health Sciences and Technology, Samsung Advanced Institute for Health Sciences and Technology, Sungkyunkwan University, Seoul, South Korea; 11 Samsung Genome Institute, Samsung Medical Center, Seoul, South Korea; 12 Department of Neurosurgery, Samsung Medical Center and Sungkyunkwan University School of Medicine, Seoul, South Korea; 13 Department of Neurosurgery, College of Medicine, Seoul National University and Seoul National University Hospital, Seoul, South Korea; 14 Department of Urology, Center for Urologic Cancer, National Cancer Center, Goyang, South Korea; 15 Department of Urology, Samsung Medical Center, Seoul, South Korea; 16 Department of Biopharmaceutical Convergence, Sungkyunkwan University, Seoul, South Korea; 17 Hong Kong Center for Neurodegenerative Diseases, InnoHK, Hong Kong SAR, China; 18 HKUST Shenzhen-Hong Kong Collaborative Innovation Research Institute, Futian, Shenzhen, China

**Keywords:** Machine learning, Glioblastoma, Temozolomide, Pharmacogenomics, Cancer genomics, Intra-tumoral heterogeneity

## Abstract

**Background:**

Although temozolomide (TMZ) has been used as a standard adjuvant chemotherapeutic agent for primary glioblastoma (GBM), treating isocitrate dehydrogenase wild-type (IDH-wt) cases remains challenging due to intrinsic and acquired drug resistance. Therefore, elucidation of the molecular mechanisms of TMZ resistance is critical for its precision application.

**Methods:**

We stratified 69 primary IDH-wt GBM patients into TMZ-resistant (*n* = 29) and sensitive (*n* = 40) groups, using TMZ screening of the corresponding patient-derived glioma stem-like cells (GSCs). Genomic and transcriptomic features were then examined to identify TMZ-associated molecular alterations. Subsequently, we developed a machine learning (ML) model to predict TMZ response from combined signatures. Moreover, TMZ response in multisector samples (52 tumor sectors from 18 cases) was evaluated to validate findings and investigate the impact of intra-tumoral heterogeneity on TMZ efficacy.

**Results:**

In vitro TMZ sensitivity of patient-derived GSCs classified patients into groups with different survival outcomes (*P* = 1.12e−4 for progression-free survival (PFS) and 3.63e−4 for overall survival (OS)). Moreover, we found that elevated gene expression of *EGR4*, *PAPPA*, *LRRC3*, and *ANXA3* was associated to intrinsic TMZ resistance. In addition, other features such as 5-aminolevulinic acid negative, mesenchymal/proneural expression subtypes, and hypermutation phenomena were prone to promote TMZ resistance. In contrast, concurrent copy-number-alteration in *PTEN*, *EGFR*, and *CDKN2A/B* was more frequent in TMZ-sensitive samples (Fisher’s exact *P* = 0.0102), subsequently consolidated by multi-sector sequencing analyses. Integrating all features, we trained a ML tool to segregate TMZ-resistant and sensitive groups. Notably, our method segregated IDH-wt GBM patients from The Cancer Genome Atlas (TCGA) into two groups with divergent survival outcomes (*P* = 4.58e−4 for PFS and 3.66e−4 for OS). Furthermore, we showed a highly heterogeneous TMZ-response pattern within each GBM patient using in vitro TMZ screening and genomic characterization of multisector GSCs. Lastly, the prediction model that evaluates the TMZ efficacy for primary IDH-wt GBMs was developed into a webserver for public usage (http://www.wang-lab-hkust.com:3838/TMZEP).

**Conclusions:**

We identified molecular characteristics associated to TMZ sensitivity, and illustrate the potential clinical value of a ML model trained from pharmacogenomic profiling of patient-derived GSC against IDH-wt GBMs.

**Supplementary Information:**

The online version contains supplementary material available at 10.1186/s13073-023-01165-8.

## Background

Isocitrate dehydrogenase wild-type (IDH-wt) glioblastoma (GBM) constitutes the most common and aggressive GBM subtype, with high inter- and intra-tumoral heterogeneity [1]. Temozolomide (TMZ), in addition to radiotherapy and surgical resection, can improve both the progression-free survival (PFS) and overall survival (OS) in newly diagnosed GBMs [2]. As a chemotherapeutic agent potentially suitable for long-term use owing to its relatively low toxicity, TMZ causes DNA damage by methylating the O^6^-position of guanine in DNA, initiating cell cycle arrest leading to cell death [3]. Despite TMZ improving survival outcomes, the recurrence rate of GBM patients after standard TMZ therapy is over 90% [4].

Identifying non-responders of TMZ in advance is especially important in neuro-oncology. To date, the promoter methylation status of O^6^-methylguanine-DNA-methyltransferase (*MGMT*), a protein that repairs the damages from TMZ, is the most widely used predictor of TMZ response in GBM [5]. However, *MGMT* methylation alone was not sufficient. Besides, it was believed that the relapsed GBM is driven by invasive GBM stem-like cells (GSCs) [6]. Under conventional treatment, invading GSCs are likely exposed to lower TMZ concentrations than the tumor cells within the contrast-enhancing tumor area highlighted by magnetic resonance imaging [7]. Therefore, the existence of residual, heterogeneous populations of GSCs explains the temporal variability of the genomic profile during GBM progression [8]. While previous studies utilized a small subset of conventional cancer cell lines to identify TMZ-resistant features [9, 10], we propose to investigate patient-derived GSCs, which might indicate treatment outcomes and reveal clinical-relevant mechanisms of drug resistance.

To identify predictive features and establish an integrative method to distinguish TMZ responder and non-responder before TMZ chemotherapy, we cultured a panel of GSCs derived from newly diagnosed treatment-naïve IDH-wt GBM patients and analyzed genomic traits and drug screening data of their early passages. Our recent work showed that these patient-derived GSCs better represent the traits of parental tumors compared to conventional cell lines [11–13]. In this study, we aimed to collect molecular profiles of the GSCs and develop a classification model to predict TMZ sensitivity in order to improve patient management.

## Methods

### Patient samples

After written informed consent was obtained, we utilized tumor specimens of patients whose first therapeutic intervention was an open surgical resection at the Samsung Medical Center in accordance with the Institutional Review Board. Overall, 128 GBM specimens (108 primary, 19 recurrent, 1 unknown) were collected from 92 GBM patients with median age at diagnosis 57 (range 29-80) including 39 females and 53 males. GBMs were diagnosed based on the World Health Organization (WHO) criteria. The methylation status of the *MGMT* promoter was assessed by methylation-specific polymerase chain reaction (PCR) after sodium bisulfite DNA modification, and the mutation of *IDH1* was detected by peptide nucleic acid-mediated clamping PCR and immunohistochemistry on the tumor tissues [14–17]. Follow-up MRI was performed at a regular interval of 2 months during treatment and 3 or 6 months interval after treatment for disease recurrence. Among the 128 specimens, 126 samples (z-score cohort, Additional file 1: Table S1) were subjected to in vitro culture of patient-derived GSCs to study relative TMZ-sensitivity (Additional file 2: Fig. S1). Within these 126 samples, TMZ-treated IDH-wt primary GBMs were selected as the main cohort (*n*=69) for downstream analysis. For intratumoral heterogeneity analysis, 18 patients with multi-sector samples (52/128) were included (multi-sector cohort). Longitudinal GBM cohort (*n* = 40 pairs) for longitudinal expression analysis included 4 (2 pairs) out of 128 samples in addition to 64 samples (32 pairs) from Wang et al. [18] and 12 samples (6 pairs) from Zhao et al. [19]. Only the pairs that were IDH1-wt in the primary (untreated) and received TMZ after the first resection were selected. WES, targeted sequencing (GliomaSCAN), and/or RNAseq were performed on the main cohort and multi-sector cohort when available. Part of the sequencing data was retrieved from our previous publications [11, 20]. Detailed information can be found in Additional file 1: Table S1.

### Isolation and short-term in vitro culture of patient-derived GSCs

Enrolled tumor specimens were enzymatically dissociated into single cells and cultured as described previously [21]. These cells were then grown in Neurobasal-A medium with N2 and B27 supplements (0.5 × each, ThermoScientific, Bartlesville, OK, USA), basic fibroblast growth factor, and epidermal growth factor (20 ng/mL each, R&D Systems, McKinley Pl NE, Minneapolis, USA). As spheres appeared in the suspension culture, they were dissociated using StemPro® (Life Technologies, Woodward St, Austin, TX, USA) and expanded by reseeding under the same suspension culture conditions. Patient-derived GSCs were negative for mycoplasma contamination, as determined using the Universal Mycoplasma Detection Kit (American Type Culture Collection, University Blvd, Manassas, USA, 30-1012K).

### TMZ sensitivity evaluation of GSCs in vitro

Patient-derived GSCs cultured in defined suspension culture conditions [21] were seeded in 384-well plates at a density of 500 cells/well with technical duplicates or triplicates. TMZ was purchased from Selleck Chemicals (Houston, TX, USA) and stored following the manufacturer’s instructions. GSCs were treated with TMZ using a fourfold and seven-point serial dilution series ranging from 500 μM to 122 nM, using a Janus Automated Workstation (PerkinElmer, Waltham, MA, USA). After 6 days of incubation at 37°C in a 5% CO_2_ humidified incubator, cell viability was assessed using an adenosine triphosphate assay system based on firefly luciferase (ATPLite™ 1step, PerkinElmer, Bridgeport Ave, Shelton, CT, USA). Cell viability was measured using an EnVision Multilabel Reader (PerkinElmer). Control wells containing only cells and vehicle (dimethyl sulfoxide) were included on each assay plate. The half maximal growth rate (GR) inhibitory concentration (GR_50_) and traditional area under the curve of the dose-response curve (AUC) were calculated using an online GR calculator [22]. These GR_50_ and AUC values were used to compute the *z*-score in a total of 126 GSC samples from GBM tumor specimens (z-score cohort, Additional file 1: Table S1) for the determination of TMZ-resistant and sensitive samples.

### DNA sequencing

Whole-exome sequencing (WES) and/or GliomaSCAN [11] were performed on the DNA fragments of the tumor and matched blood. For WES, exonic DNA was captured by Agilent SureSelect Kit. GliomaSCAN is a massive parallel targeted sequencing protocol that covers exons of selected glioma-associated genes. Pair-end sequencing was sequenced on Illumina HiSeq 2000 instrument. FASTQ data was mapped to human genome reference (hg19) using Burrows-Wheeler Aligner [23]. Duplicates were marked by Picard and alignments were sorted by SAMtools [24].

### Somatic mutation detection

SAVI2 [18] was used for identifying somatic mutations from WES and targeted sequencing (GliomaSCAN) [11]. From the SAVI2 report, nonsynonymous somatic mutations with tumor variant allele frequency (VAF) higher than 5% and matched blood VAF equal to 0% were selected. Selected GBM driver genes were used in the following analysis. For epidermal growth factor receptor variant III (EGFRvIII), a sample was determined EGFRvIII-positive if two or more reads skipped exon 2–7 from the transcriptomic data.

### Copy number alteration by WES and GliomaSCAN

We used the ngCGH python package (version 0.4.4) [25] to generate estimated copy number alterations (CNAs) in a tumor specimen compared with its matched blood control. Gene-level read counts were calculated in both tumor and matched control. The output value from the package, which is the median-centered log2 ratio of tumor and normal sample, was used to define copy number status. If the value was above 0.5, the gene was annotated as “gain,” and “amplification” if above 1.58. Similarly, a value lower than −0.5 and −1.58 was labeled as “loss” and “deletion,” respectively. However, in the case of EGFR, GliomaSCAN’s copy number result was less accurate and therefore 0.3 and 1.58 were used as cut-offs to increase compatibility with WES results. The CNA result from WES data had the highest priority followed by CNA called from GliomaSCAN and RNA sequencing (RNA-seq).

### CNA estimation by RNA sequencing

For samples with RNA-seq data but without WES, we estimated the CNAs from RNA-seq by adopting the CNAPE method [26] with several modifications. Briefly, we used XGBoost [27] to train our model instead of LASSO (Least Absolute Shrinkage and Selection Operator) regression, and also took the KEGG (Kyoto Encyclopedia of Genes and Genomes) pathway gene set into consideration instead of only the STRING (Search Tool for the Retrieval of Interacting Genes/Proteins) protein-protein interaction. Then we used 38 samples with matched WES and RNA-seq data from our dataset to calibrate the cut-off values of normal, gain, and amplification (or loss and deletion) via optimizing F-score. Details can be found in Additional file 3: Supplementary Methods.

### RNA-Seq data processing and gene expression marker identification

Sequencing reads were mapped to human genome reference (hg19) by the STAR (Spliced Transcripts Alignment to a Reference) pipeline [28]. Read counts were then calculated using featureCounts [29]. To identify genes that have conserved expression profiles between GSCs and the matched initial tumor tissues, RNA sequencing analyses were carried out on 12 matched GSC-tissue pairs to calculate the Spearman correlation coefficient for each gene based on log2-transformed raw read counts. A Gaussian mixture model was then used to separate conserved genes from the non-conserved genes (Additional file 2: Fig. S2). The conserved genes (with Spearman correlation coefficient > 0.177) were subsequently investigated by differential gene expression analysis via DESeq2 R package [30] on RNA-seq data of tumor tissues from 12 TMZ-resistant and 22 TMZ-sensitive samples (evaluated by the above-mentioned in vitro TMZ screening). Principal component analysis was performed on these 34 samples to detect potential batch effects (Additional file 2: Fig. S3a, b). To make sure the marker genes are more reliable, we used stringent cut-offs (log_2_ fold change > 2.5, adjusted *P* < 0.01) for identifying differentially expressed genes resulting in four TMZ-resistant markers (Additional file 2: Fig. S3c). To measure the level of gene expression, read counts were converted to Reads Per Kilobase per Million mapped reads (RPKM), followed by log2 transformation and quantile normalization.

### GBM subtyping

We performed single sample gene set enrichment analysis (ssGSEA) using the GBM subtype gene sets defined by Wang et al. [31] on the RNA-seq samples. The enrichment scores for each subtype were normalized across samples. The subtype with the highest normalized enrichment score was selected as the activated subtype for each sample.

### TCGA data

Transcriptomic data of the TCGA cohort was downloaded directly from Broad GDAC Firehose (normalized RNAseqv2 RSEM, https://gdac.broadinstitute.org/). Mutation and CNA data were downloaded from cBioPortal. Clinical data was downloaded from the original publication by Ceccarelli et al. [1] and cBioPortal [32].

### Modeling TMZ efficacy predictor (TMZep)

The XGBoost classifier [27] was trained to separate TMZ responder from non-responder based on genomic and transcriptomic profiles. A total of 25 features, including methylation status of *MGMT* promoter, single-nucleotide variants (SNVs), CNAs, and expression levels of selected genes, were incorporated to train a machine-learning model, based on samples in the main cohort (*n* = 69). To address the issue of missing values, we first performed data imputation: for binary features, missing values were replaced by 0.5; for continuous features, missing values were imputed by KNNImputer [33] (*K*=5). The imputed data was used to train the XGBoost model (python xgboost v0.90), where 50 decision trees with a tree depth of no more than 3 were constructed under the learning rate of 0.74 and the subsampling ratio of 0.35 for each boosting iteration. The above hyperparameters were selected via optimization of (1) AUC score in 5-fold cross-validation; (2) capability of stratifying patients with different survival outcomes in the training set; and (3) biological significance of prioritized features. In the final model, we used 0.6 as the probability cutoff to segregate two risk groups. Furthermore, we added L2 regularization to the cost function to control overfitting and enhance the generalization ability of our model for unseen data. Lastly, the area under receiver operating characteristic curve (AUC) score was used to measure the model’s performance.

### Statistical analysis

*T*-test, Wilcoxon rank-sum test, Spearman’s rank correlation coefficient test, and Fisher’s exact test were used to conduct different statistical analyses. Survival analyses were performed using the Kaplan–Meier method and the Cox proportional hazards regression method. Patients who were alive at the last known follow-up were considered censored in these analyses. Hazard ratios (HR) and their 95% confidence intervals (CIs) were calculated. Statistical analyses were conducted using Python (v.3.8) and R (3.6.3) software.

## Results

### In vitro screening using patient-derived GSCs reflects personalized TMZ efficacy

**Fig. 1:**
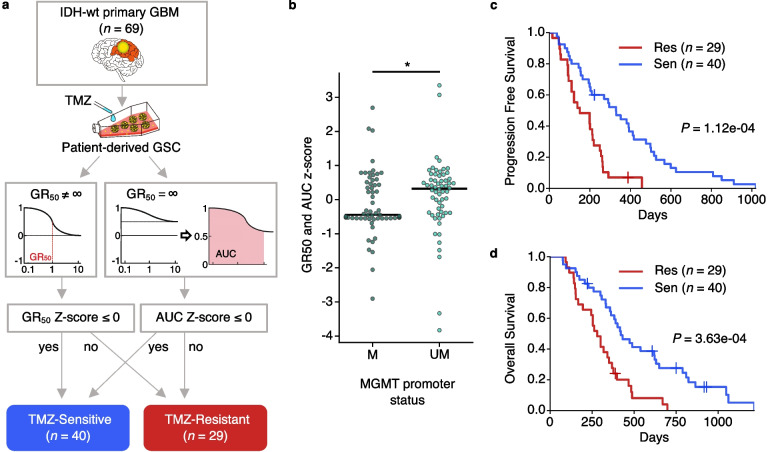
In vitro TMZ screening of patient-derived glioblastoma (GBM) stem cell (GSC) predicts GBM prognosis. **a** TMZ sensitivity determination pipeline for patient-derived GSC. GR: growth rate; AUC: area under the conventional drug response curve; GSC: Glioblastoma Stem Cell; TMZ: temozolomide. **b** GR50 and AUC *z*-score comparison between *MGMT* methylated and un-methylated samples. Wilcoxon rank sum tests were performed for *p*-values (* *P* < 0.05). **c**, **d** Comparison of progression-free survival (**c**) and overall survival (**d**) between the TMZ-resistant (red) and TMZ-sensitive (blue) patients. The cohort was determined by TMZ screening in panel **a**. Sen, TMZ-sensitive; Res, TMZ-resistant. *P*-values were calculated by logrank test

To evaluate GBM’s response to TMZ, we performed in vitro TMZ cytotoxicity assays in short-term (6 days) cultured patient-derived GSCs (*n* = 69, main cohort) obtained from surgically resected IDH-wt primary GBM specimens. Since conventional metrics such as the effective concentration at 50% (IC_50_) or maximum inhibition % (E_max_) highly depends on cell division rate obscuring accurate sensitivity prediction, we adopted GR inhibition metrics, which are independent of division number and therefore superior to conventional metrics for assessing the effects of drugs in fast dividing cells [34]. We calculated GR_50_ values for each sample, and for those with infinite GR_50_ values, we measured conventional AUC values. By calculating *Z*-scores for GR_50_ and AUC, we divided our samples into TMZ-Sensitive and TMZ-Resistant groups (Fig. 1a). As expected, *MGMT* promoter methylation was observed to be related to *Z*-scores of GR_50_ and AUC values (Fig. 1b, Wilcoxon rank sum test *P* = 0.018) [35].

Strikingly, TMZ-Resistant (*n* = 29) and TMZ-Sensitive (*n* = 40) groups defined from in vitro sensitivity were highly predictive of survival outcomes for patients who were under a TMZ-based treatment regimen (Fig. 1c and d; PFS, *P* = 1.12e−4; OS, *P* = 3.63e−4; by log-rank test). Notably, the above-defined in vitro sensitivity surpasses the well-known *MGMT* promoter methylation status in predicting the patient prognosis (Additional file 2: Fig. S4a). Additionally, a Cox-regression multivariate survival analysis considering age, gender, extent of resection, and *MGMT* promoter methylation revealed that in vitro TMZ sensitivity and the extent of resection were independent factors associated with PFS and OS, while *MGMT* promoter methylation being related to in vitro sensitivity (Additional file 2: Fig. S3b, Fisher’s exact test *P* = 0.0156) was marginally significant (Table 1). Collectively, these data reflect the reliability of our preclinical TMZ testing system for assessing clinical response to TMZ in patients newly diagnosed with IDH1-wt GBM.

**Table 1:** Cox-regression analysis of survival outcomes in the 69 cases screened with TMZ in vitro

Variables (number of samples)	PFS						OS					
	Univariate			Multivariate			Univariate			Multivariate		
	HR	95% CI	***P***	HR	95% CI	***P***	HR	95% CI	***P***	HR	95% CI	***P***
**Age** **(<50 years,** ***n*** **= 13) vs. (≥50 years,** ***n*** **= 56)**	0.803	0.425–1.52	0.500				0.857	0.451–1.63	0.638			
**Gender** **Male (*n*** **= 34) vs. Female (*n*** **= 35)**	1.11	0.671–1.84	0.681				0.881	0.524–1.49	0.636			
***MGMT*** **promoter methylation** **UM (*n*** **= 35) vs. M (*n*** **= 33)**	2.26	1.32–3.88	0.003	1.71	0.973–3.00	0.0622	1.708	0.992–2.94	0.0535			
**Extent of resection** **GTR (*n*** **= 35) vs. STR (*n*** **= 29)**	1.97	1.18–3.30	9.56e−3	2.00	1.19–3.38	9.10e−3	1.722	1.02–2.92	0.0432	2.02	1.17–3.49	0.0114
**In vitro TMZ sensitivity** **Resistant (*n*** **= 29) vs. Sensitive (*n*** **= 40)**	2.75	1.55–4.91	5.71e−4	2.45	1.33–4.52	4.06e−3	2.537	1.45–4.45	1.18e−3	2.90	1.61–5.19	3.6e−4

*PFS* progression-free survival, *OS* overall survival, *HR* hazard ratio, *CI* confidence interval, *UM* unmethylated, *M* methylated, *GTR* gross total resection, *STR* subtotal resection

### Genomic analysis reveals somatic mutational landscape of TMZ-resistant and sensitive groups

**Fig. 2:**
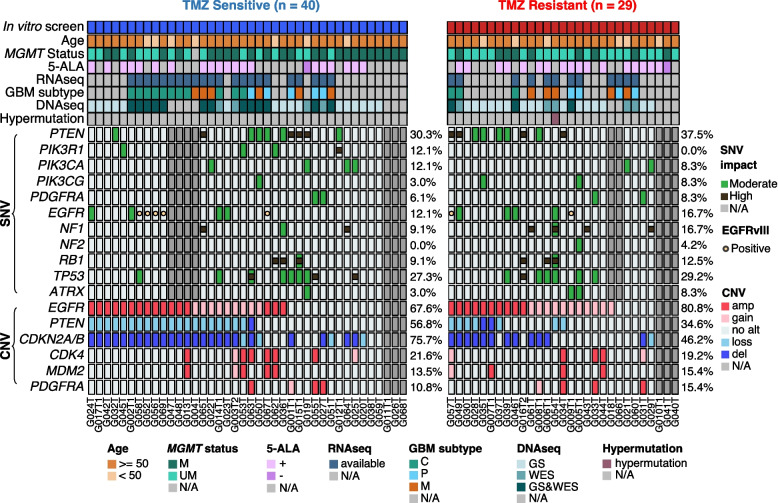
Somatic mutational landscape of the main cohort. TMZ sensitivity was determined based on in vitro TMZ screening. The single nucleic variants (SNV) including point mutations, short insertion/deletions, and copy number amplifications and deletions of selected GBM driver genes were included. Alteration frequencies are shown on the right side. When available, copy number alteration results were inferred from the methods in the following order; WES (highest priority), Gliomascan, and RNAseq. EGFRvIII was identified from RNA-seq data. Moderate: missense or inframe deletion; high: frameshift, stop gained, splice donor or splice acceptor; M, methylated; UM, unmethylated; N/A, not available; C, classical; P, proneural; M, mesenchymal; GS, Gliomascan; WES, whole exome sequencing

To identify genetic factors contributing to TMZ response, we explored somatic genomic alterations in the TMZ-resistant and sensitive groups in our main cohort. WES and/or GliomaSCAN on 57 tissue specimens (with matched blood controls) and RNA-seq on 34 tissue specimens were either newly performed or downloaded from previous publications [11, 20] (Additional file 1: Table S1). Somatic SNVs and short insertions/deletions were identified by SAVI2 [18] (Additional file 1: Table S2). A sample was labeled as hypermutated if the total number of somatic mutations was over 350 by WES. CNAs were calculated from WES, GliomaSCAN, or were predicted from RNA-seq by CNAPE [26] (Additional file 1: Table S2-3, Additional file 2: Fig. S5-S6, and Additional file 3: Supplementary Methods). Variants with VAF over 5% and CNAs in previously reported GBM driver genes, together with EGFRvIII (Additional file 1: Table S4) and the expression-based GBM subtyping were shown in Fig. 2. Overall, no significant genomic difference was observed between the responder and non-responder groups. Yet, mesenchymal/proneural subtype and somatic mutations in genes including *NF1*, *NF2*, and *PTEN* were more often observed in TMZ-resistant samples, while *PIK3R1* somatic mutations were slightly more frequent in TMZ-sensitive samples (Fig. 2). These observations indicate that GBM’s response to TMZ might be determined by the combination of multiple factors but not by single ones.

### Transcriptomic sequencing reveals marker genes of TMZ resistance

**Fig. 3:**
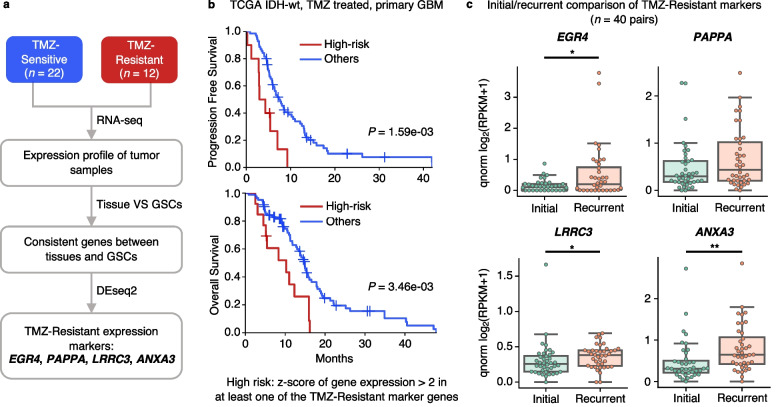
Elucidation of expression markers associated with the resistance to temozolomide in patients with IDH-wt primary glioblastoma (GBM). **a** TMZ-resistant expression marker identification using RNA sequencing (*n* = 34). **b** Progression-free survival (upper panel) and overall survival (bottom panel) of IDH-wt, TMZ-treated primary GBM from the TCGA. High risk, *z*-score of gene expression > 2 in at least one of the TMZ-resistant marker genes (*n* = 13); others, the rest (*n* = 83). **c** Comparison of the expression level of the TMZ-Resistant expression markers in the initial and recurrent paired samples from the longitudinal sequencing cohort with 40 IDH-wt, TMZ-treated primary GBMs. Each gray line connects the gene expression level in one initial and recurrent pair. Wilcoxon rank sum tests were performed for *p*-values (* *P* < 0.05, ** *P* < 0.01). qnorm, quantile normalized

To identify marker genes of TMZ response, we first separated conserved and non-conserved gene expression between GSCs and the initial tumor tissue through a Gaussian mixture model (Additional file 2: Fig. S2). The conserved genes were then used to perform differential gene expression analysis between tissue RNA-seq data of TMZ-resistant (*n* = 12) and sensitive (*n* = 22) samples. Principal component analysis on these 34 samples showed slight clustering by GBM subtype (proneural vs. mesenchymal/classical) but by no other factors including age, gender, and MGMT promoter methylation status (Additional file 2: Fig. S3a, b). We identified four genes (*EGR4*, *PAPPA*, *LRRC3*, and *ANXA3*) significantly up-regulated in the TMZ-resistant group (Fig. 3a, Additional file 2: Fig. S3c, log_2_ fold change > 2.5, adjusted *P* < 0.01). To explore the prognostic value of the TMZ-resistant marker genes, we extracted 96 RNA-seq available TMZ-treated IDH-wt primary GBM patients from the TCGA dataset and classified them into high-risk and low-risk groups based on the expression of these four genes. Notably, the high-risk group had significantly worse PFS (*P* = 1.59e−03 by log-rank test) and OS (*P* = 3.46e−03 by log-rank test, Fig. 3b), compared to that of the low-risk group.

To further investigate the expression change of these genes before and after TMZ treatment, we integrated a total of 40 paired RNA-seq data of initial and matched TMZ-treated recurrent IDH-wt GBM samples [18, 19]. As shown in Fig. 3c, the expression level of TMZ-resistant markers increased in the recurrent samples compared to the initial, suggesting that the TMZ-resistant marker-expressed cell population survived TMZ treatment and expanded in the recurrent GBM.

### A machine learning (ML) approach for integrating key features to predict TMZ response of IDH1-wt GBM

**Fig. 4:**
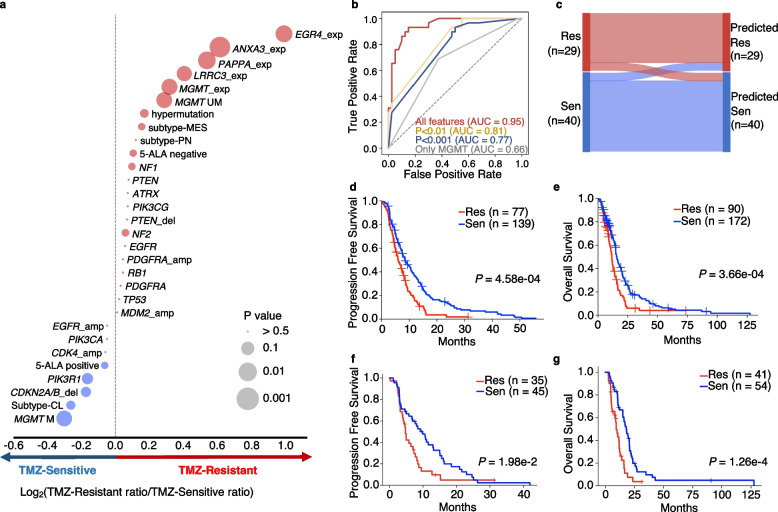
Machine learning from the combined genomic and expression features predicts patient prognosis. **a** Bubble plot showing the trends of features in terms of TMZ-resistant and TMZ-sensitive. The bubble size indicates *P*-value, the color and location of the bubble indicate the log2 of TMZ-resistant ratio/TMZ-Sensitive ratio value. If the log2 TMZ-Resistant ratio/TMZ-Sensitive ratio value is positive, the bubble is colored in red, and if negative, it is colored in blue. Copy number gain and loss were not counted in this plot. del: deletion, amp: amplification, exp: expression, subtype: GBM subtype; CL, classical; PN, proneural; MES, mesenchymal; M, methylated; UM, unmethylated. *P* values on gene expression and *MGMT* fusion bubbles are by t-test, the rest are by Fisher’s exact test. **b** ROC curve in the training set (*n* = 69). All features include 25 features shown in **a**. *P* < 0.01, using features that are *P* < 0.01 in **a** (gene expression of *ANXA3*, *PAPPA*, *EGR4*; AUC: 0,81); *P* < 0.001, using features that are *P* < 0.001 in **a** (*ANXA3* expression; AUC: 0.77); *MGMT*, only using *MGMT* promoter status as prediction feature. **c** Sankey diagram showing confusion matrix of resistant and sensitive samples in the training dataset. Sen, TMZ-sensitive; Res, TMZ-resistant. **d**, **e** Survival curves of TCGA IDH-wt, TMZ-treated primary GBM samples which the TMZ response has been predicted by machine learning. P-values were calculated by logrank test. **f**, **g** Survival curves of mesenchymal TCGA samples separated by predicted TMZ response. *P*-values were computed by log-rank test

Figure 4a presents the overall relevance of the genomic, transcriptomic, and other features on TMZ response. Along with the expression of four TMZ-resistant markers, *MGMT* expression, *MGMT* promoter methylation status, hypermutation status, GBM subtype, somatic mutations, and CNAs identified from the main cohort, we added 5-aminolevulinic acid (5-ALA) tendency [36] as another feature (Additional file 1: Table S1). In order to integrate these features for patient evaluation, we constructed an XGBoost classifier to identify the TMZ response of a patient as TMZ-resistant or TMZ-sensitive. Among the 30 features shown in Fig. 4a, 25 features were used to train the machine learning model in the main cohort, excluding *NF2* mutation, hypermutation, 5-ALA positive and 5-ALA negative which were not available in the TCGA testing cohort (Additional file 1: Table S5). Compared with *MGMT* promoter status as the only feature, adding other features provided more information for recognition of TMZ non-responders (Fig. 4b). Notably, the top five informative features from the model were the expression level of *ANXA3* and *LRRC3*, proneural subtype, *ERG4* and *MGMT* expression (Additional file 2: Fig. S7a). In addition, incorporating the four expression markers together with other features achieved a stronger discrimination power compared to the presence of just an individual marker (Additional file 2: Fig. S7b). Within the training cohort (main cohort), a prediction of 88.4% (61 out of 69) of the samples matched the in vitro TMZ-response (Fig. 4c). We then tested our model in an independent cohort with 262 IDH-wt, TMZ-treated primary GBM patients from TCGA (inclusive of the 96 RNA-seq available patients from Fig. 3b). Importantly, patients predicted to be TMZ-resistant by the classifier had significantly worse PFS (Fig. 4d, *P* = 4.58e−04 by log-rank test) and OS (Fig. 4e, *P* = 3.66e−04 by log-rank test) validating the power of our model to predict prognostic outcome in patients treated by TMZ. Moreover, we investigated the survival difference across four subtypes (classical, proneural, neural, and mesenchymal) in the TCGA cohort and no significant segmentation was observed for PFS (*P* = 0.531 by multivariate log-rank test) and OS (*P* = 0.412 by multivariate log-rank test) in Additional file 2: Fig. S8a, which is expected and compatible with the observations previously reported by the TCGA group. We further correlated the four GBM subtypes with the TMZ response predicted from our machine learning model. Notably, the mesenchymal subtype is associated with TMZ resistance (*P* = 0.039 by Fisher exact test, Additional file 2: Fig. S8b). Among the mesenchymal cases, the resistant group demonstrated worse PFS (*P* = 1.98e−02 by log-rank test, Fig. 4f) and OS (*P* = 1.26e−04 by log-rank test, Fig. 4g), compared to that of the sensitive group, highlighting the value of our model to unveil new responders/non-responders within the subtypes. In addition, when compared to using only *MGMT* promoter methylation status in the TCGA dataset (*n* = 203), our model integrating multiple features provided a better way to segregate patients with different outcomes of both PFS and OS (Additional file 2: Fig. S9). Within *MGMT* methylated group, our model identified a limited number of high-risk resistant cases with worse PFS and OS (Additional file 2: Fig. S9c). Furthermore, to facilitate the use of our model, we designed a freely accessible website named TMZep that provides the function for evaluating potential TMZ response for GBM patients (http://www.wang-lab-hkust.com:3838/TMZEP) [37]. Users can input patient’s data on part or all of the 25 features to the website, which will evaluate the potential TMZ treatment response of the corresponding GBM patient.

### Multi-sector TMZ screening underlines intratumoral heterogeneity in drug responsiveness

**Fig. 5:**
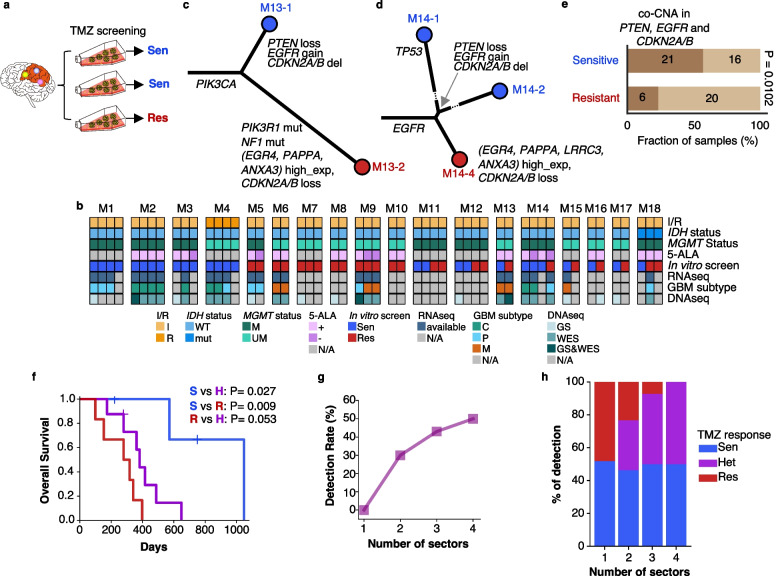
TMZ screening in multi-sector samples underscores intra-tumor heterogeneity of drug response. **a** Experimental design for screening multi-sector samples. Sen, TMZ-sensitive; Res, TMZ-resistant. **b** Molecular features of multi-sector samples. I, initial tumor; R, recurrent tumor; WT, wild-type; mut, mutant; M, methylated; UM, unmethylated; N/A, not available; C, classical; P, proneural; M, mesenchymal. **c**, **d** Phylogenetic trees of somatic mutation evolution in multi-sector samples from **c** patient M13 and **d** patient M14. The length of the branch is relative to the number of mutations. Dashed lines indicate a relatively larger number of mutations that cannot be scaled for visualization. Indicated alterations are GBM driver alterations and RNA expression of TMZ-resistant markers. amp, amplification; del, deletion; higher_exp, higher transcriptomic expression compared to other sample/samples. Blue, TMZ-sensitive; red, TMZ-resistant. **e** Comparison of Concurrent CNAs in *PTEN*, *EGFR*, and *CDKN2A/B* in the main cohort. *P* value by Fisher’s exact test. **f** Overall survival difference in patients with multi-sector samples identified as S, all sensitive (M1~M4); H, heterogeneous (M11~M18); R, all resistant (M5~M10). *P*-values calculated by logrank test. **g** Detection rate of TMZ heterogeneity by the number of multi-sector samples. **h** Relative distribution of TMZ response by the number of multi-sector samples

Intratumoral heterogeneity (ITH) is a key factor that causes therapeutic resistance and recurrence in GBM [38, 39]. To reveal the impact of ITH on TMZ treatment, we compiled 52 GBM tumor tissue specimens from 18 patients, where each patient had 2 to 4 multi-sector samples taken from the same patient (Multi-sector cohort, Additional file 1: Table S1). Of the Multi-sector cohort, 15 tissues and patients overlapped with the main cohort. The three additional patients were patients with recurrent GBM, IDH mutant GBM, or IDH-wt primary GBM without TMZ treatment. We performed in vitro TMZ screening of the multi-sector GSCs (Fig. 5a) followed by WES in 19 samples and RNA-seq in 26 samples. Interestingly, almost half of the patients (8/18) carried both TMZ-resistant and TMZ-sensitive tumor samples (Fig. 5b and Additional file 2: Fig. S10). We termed these patients as TMZ-ITH, which harbored heterogeneous GSCs within one tumor in terms of in vitro TMZ treatment response. We confirmed several TMZ-associated factors identified earlier in this study by comparing the molecular signatures of multi-sectors. In particular, the TMZ-resistance markers were upregulated in the resistant sectors of M13 and M14 (Fig. 5c, d, Additional file 2: Fig. S11a-b). Meanwhile, a combination of *PTEN* loss, *EGFR* gain, and deeper deletion of *CDKN2A/B* was observed specifically in the sensitive sectors of these two patients (Fig. 5c, d, and Additional file 2: Fig. S11c). Motivated by this observation, we checked the concurrent CNAs in *PTEN*, *EGFR*, and *CDKN2A/B* back in our main cohort and found that it was significantly more frequent in TMZ-sensitive samples (Fisher’s exact *P* = 0.0102, Fig. 5e), while each individual factor did not have statistical significance.

Back to the ITH analysis, eight TMZ-ITH patients had comparable survival time with patients harboring only TMZ-resistant sectors and significantly worse survival time than patients with only TMZ-sensitive sectors (OS, *P* = 0.027; PFS, *P* = 0.015; by log-rank test), indicating that although the TMZ treatment achieved the particular effect by eliminating a sensitive group of tumor cells, the resistant GSCs might quickly lead to tumor relapse (Fig. 5f and Additional file 2: Fig. S12). This observation underscores the importance of careful consideration of ITH via multi-sector evaluation before treatment delivery.

Since the number of sectors analyzed from a tumor may influence the possibility of TMZ-ITH detection, we evaluated the optimal number of sectors to observe TMZ-ITH in a patient. We demonstrated that when two sectors were taken from a tumor, the TMZ-ITH detection rate was around 30% (17/56), followed by 43% (12/28) with three sectors, and 50% (3/6) with four sectors (Fig. 5g). Interestingly, while the TMZ-ITH detection increases with multi-sector number, purely resistant groups decreased but not the purely sensitive patients, underscoring the existence of good responders of TMZ treatment (Fig. 5h).

## Discussion

**Fig. 6:**
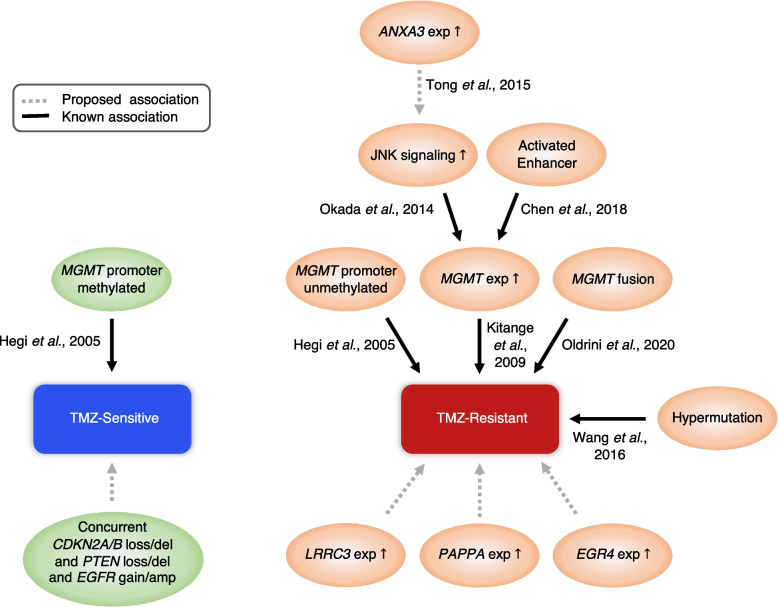
Summarizing scheme for the known and newly identified molecular features associated to TMZ response in GBM. References supporting the associations are shown next to the arrows [18, 35, 41–45]. Features with gray dotted lines are the proposed association from this study. Small arrows pointing upward inside the bubbles indicate activated signaling or highly expressed

To date, TMZ is the major standard chemotherapeutic agent for primary GBM treatment. However, recent studies do not support the indiscreet use of TMZ because of its side effects [2, 40]. Moreover, treatment outcome significantly differs among patients due to personalized genetic background and various tumor microenvironment [35]. Therefore, precision identification of TMZ responders is in urgent need to optimize TMZ-related treatment and benefit patients. In this study, we demonstrated that in vitro screening of TMZ on patient-derived GSCs, which distinguishes TMZ-resistant and sensitive groups, is related to prognosis, reflecting TMZ efficacy in patients. However, this option has several challenges: culturing GSCs may not be always successful, is of high cost, and is not yet available widely. To develop a more easily accessible tool for TMZ-sensitivity prediction, we performed multi-omic analysis on the TMZ-resistant and sensitive GBM specimens. Transcriptomic comparison between these two groups revealed four TMZ-resistant markers, i.e., *EGR4*, *PAPPA*, *LRRC3*, and *ANXA3*. Along with these markers, we investigated the association of TMZ sensitivity and other molecular features such as somatic mutations and CNAs. Systematically integrating these features, we constructed a machine learning-based model which was able to classify IDH-wt primary GBM patients into TMZ-resistant and sensitive groups with high prognostic value. In addition, we demonstrated the dramatic impact of ITH by evaluating multi-sector samples from the same patients. Noticeably, patients with all sectors sensitive to TMZ had the most optimistic treatment outcome. Meanwhile, the multi-sector study validated important features associated with TMZ response. Together, we proposed and summarized several new TMZ response-associated features in addition to the well-known factors in this study (Fig. 6).

The expression level of the four TMZ-resistant markers predicted poor survival not only in our cohort but also in an independent IDH-wt GBM cohort extracted from the TCGA dataset. In addition, higher expression of these genes was observed in the recurrent GBMs and the TMZ-resistant sectors of the TMZ-ITH patients, highlighting the role of these genes in contributing to TMZ resistance. Although further studies will be needed to investigate the underlying mechanisms of these genes, it was reported that the *ANXA3* gene drives tumor growth through the c-Jun N-terminal kinase (JNK) pathway [41]. Compelling evidence indicates a role for JNKs in the maintenance of GSCs [46] and regulating TMZ resistance through *MGMT* expression [42].

On the other hand, we observed co-occurrence of *CDKN2A/B* loss/deletion, *PTEN* loss/deletion, and *EGFR* gain/amplification more frequently in the TMZ-sensitive samples from the main cohort and the multi-sector cohort, while each single feature was not statistically significant. According to the fifth edition of the WHO classification of tumors of the central nervous system (CNS), *EGFR* amplification and +7/−10 copy number changes (*PTEN* in chromosome 10) are the parameters for Glioblastoma IDH-wt diagnosis, while *CDKN2A/B* homozygous deletion is the parameter to diagnose IDH-mutant astrocytoma as WHO CNS grade 4 [47], suggesting that the CNAs in the three genes are related to more aggressive CNS tumors. While the prognostic value of EGFR alterations within GBMs is still controversial, some studies have reported its association with better outcomes [48–50]. Hobbs et al. reported that high EGFR-amplified GBMs had a favorable response to TMZ compared to no or low-amplified GBMs [48]. They speculated that EGFR-amplified GBMs may have higher genome fragility making them more susceptible to DNA damage induced by TMZ [48]. Yet how the concurrent *CDKN2A*, *PTEN*, and *EGFR* CNAs affect TMZ-response in GBM is unknown and the investigation therefore belongs to the realm of future work.

Considering that single features showed limited power to predict TMZ efficacy, we developed a machine learning model to integrate many features to predict TMZ responders. The model outperforms single feature models, which would assist the improvement of TMZ treatment on GBM patients. However, the model was validated only in the TCGA dataset, so the chance of over-fitting cannot be fully ruled out. Prediction results in other datasets may vary due to various reasons such as the clinical settings of the hospital and the treatment of the patients. Therefore, our model is yet preliminary to be directly applied to practice, and evaluation on a larger additional independent cohort would be necessary in future studies. In addition, we identified a new patient group with TMZ resistance within the MGMT methylated group, but due to the small sample size of these patients, additional follow-up is necessary to confirm these results.

Although more accessible, our model’s prediction using multi-omic features is still less accurate than in vitro screening, partially due to that the current markers may not be complete, and more markers such as non-coding genomes or epigenomic features remain to be discovered. In addition, the features may not be independent, so more advanced multi-omics integration methods could be applied to reveal interactions between different data layers and further improve the model’s robustness. Moreover, utilizing single-cell sequencing or cell-type deconvolution technologies (e.g., CIBERSORT, xCell) to assess the TME composition as well as resistant and sensitive tumor samples could be promising future directions to further demonstrate how concordant the cell-type compositions can affect treatment outcomes.

## Conclusions

In summary, we demonstrated that in vitro TMZ screening of patient-derived GSCs can reflect treatment outcomes in IDH-wt GBM patients under the standard Stupp therapy (radiotherapy with concomitant TMZ followed by adjuvant TMZ). Genomic and transcriptomic characterization revealed MGMT promoter methylation status, hypermutation, and the expression of MGMT, EGR4, ANXA3, PARPA, and LRRC3, together with other features, as relevant molecular predictors of TMZ response for IDH-wt GBMs. The machine learning model TMZep [37] for predicting TMZ efficacy from pharmacogenomic data integration provided an easily assessable computational tool to facilitate a more selective treatment towards the disease.

## Supplementary Information

**Additional file 1: Table S1.** Clinical annotations of 128 GBM samples used in the study. **Table S2.** Variants identified by WES and GliomaSCAN. **Table S3.** Copy number alteration by WES and/or RNA-seq. **Table S4.** EGFRvIII detection in RNA-seq available samples. **Table S5.** Input file used for machine learning training.

**Additional file 2: Fig. S1.** Timeline of sample acquisition, sequencing, *in vitro* culture and TMZ screening. **Fig. S2.** Gaussian Mixture Model used to identify genes with the same expression profile between patient-derived cells (PDCs) and tumor tissues. **Fig. S3.** Principal Component Analysis and differentially expressed gene analysis on 34 tissue RNA-seq samples. **Fig. S4.** Association of MGMT promoter methylation status to survival and *in vitro* TMZ screening in the main cohort. **Fig. S5.** Copy number estimation by GliomaSCAN. **Fig. S6.** Copy number estimation by RNA-seq. **Fig. S7.** Machine learning model feature importance. **Fig. S8.** Correlations between GBM subtypes and TMZ response. **Fig. S9.** Comparison of survival prediction in TCGA cohort. **Fig. S10.** Genomic landscape of multi-sector samples. **Fig. S11.** TMZ-resistant marker expression and CNV comparison in patient M13 and M14. **Fig. S12.** Progression free survival difference in patients with multi-sector samples.

**Additional file 3.** Supplementary methods.

## Data Availability

All newly sequenced datasets used and analyzed during the current study (GSC RNA-seq, GBM tissue RNA-seq, GBM WES, GBM GliomaSCAN) have been deposited in the European Genome-phenome Archive (https://ega-archive.org/) under accession EGAS00001006989 (https://ega-archive.org/studies/EGAS00001006989) [51]. Previously published data can be downloaded from the Sequence Read Archive (SRP074425 [18]: GBM paired RNA-seq; PRJNA482620 [19]: GBM paired RNA-seq) and the European Genome-phenome Archive (EGAS00001002515 [11]: GSC RNA-seq, GBM tissue RNA-seq, GBM WES, GBM GliomaSCAN; EGAS00001001880 [20]: GBM tissue RNA-seq, GBM WES; EGAS00001001800, EGAS00001000579, EGAD00001001113, and EGAD00001001424 [18]:GBM paired RNA-seq). More details on previously published samples and the corresponding archive can be found on Table S1. Relevant scripts regarding main results and model training are available at https://github.com/WangLabHKUST/TMZscript [52].

## Abbreviations

TMZ: Temozolomide
GBM: Glioblastoma
IDH-wt: Isocitrate dehydrogenase wild-type
ML: Machine Learning
GSCs: Glioma stem-like cells
PFS: Progression-free survival
OS: Overall survival
TCGA: The Cancer Genome Atlas
MGMT: O^6^-methylguanine-DNA-methyltransferase
GR_50_: Half maximal growth rate inhibitory concentration
AUC: Area under the curve of the dose-response curve
WES: Whole-exome sequencing
VAF: Variant allele frequency
EGFRvIII: Epidermal growth factor receptor variant III
RNA-seq: RNA sequencing
CNAs: Copy number alterations
RPKM: Reads per kilobase per million mapped reads
ssGSEA: Single sample gene set enrichment analysis
GR: Growth rate inhibition
5-ALA: 5-Aminolevulinic acid
ITH: Intratumoral heterogeneity
