# Supplementary material for: Pharmacogenomic profiling reveals molecular features of chemotherapy resistance in IDH wild-type primary glioblastoma
Source: Genome Med. 2023 Mar 13;15:16. doi: 10.1186/s13073-023-01165-8 (PMC10010007; doi:10.1186/s13073-023-01165-8)
Supplement: Supplementary file 2 — Additional file 2: Fig. S1. Timeline of sample acquisition, sequencing, in vitro culture and TMZ screening. Fig. S2. Gaussian Mixture Model used to identify genes with the same expression profile between patient-derived cells (PDCs) and tumor tissues. Fig. S3. Principal Component Analysis and differentially expressed gene analysis on 34 tissue RNA-seq samples. Fig. S4. Association of MGMT promoter methylation status to survival and in vitro TMZ screening in the main cohort. Fig. S5. Copy number estimation by GliomaSCAN. Fig. S6. Copy number estimation by RNA-seq. Fig. S7. Machine learning model feature importance. Fig. S8. Correlations between GBM subtypes and TMZ response. Fig. S9. Comparison of survival prediction in TCGA cohort. Fig. S10. Genomic landscape of multi-sector samples. Fig. S11. TMZ-resistant marker expression and CNV comparison in patient M13 and M14. Fig. S12. Progression free survival difference in patients with multi-sector samples. [file 13073_2023_1165_MOESM2_ESM.pdf]

Supplementary Figures

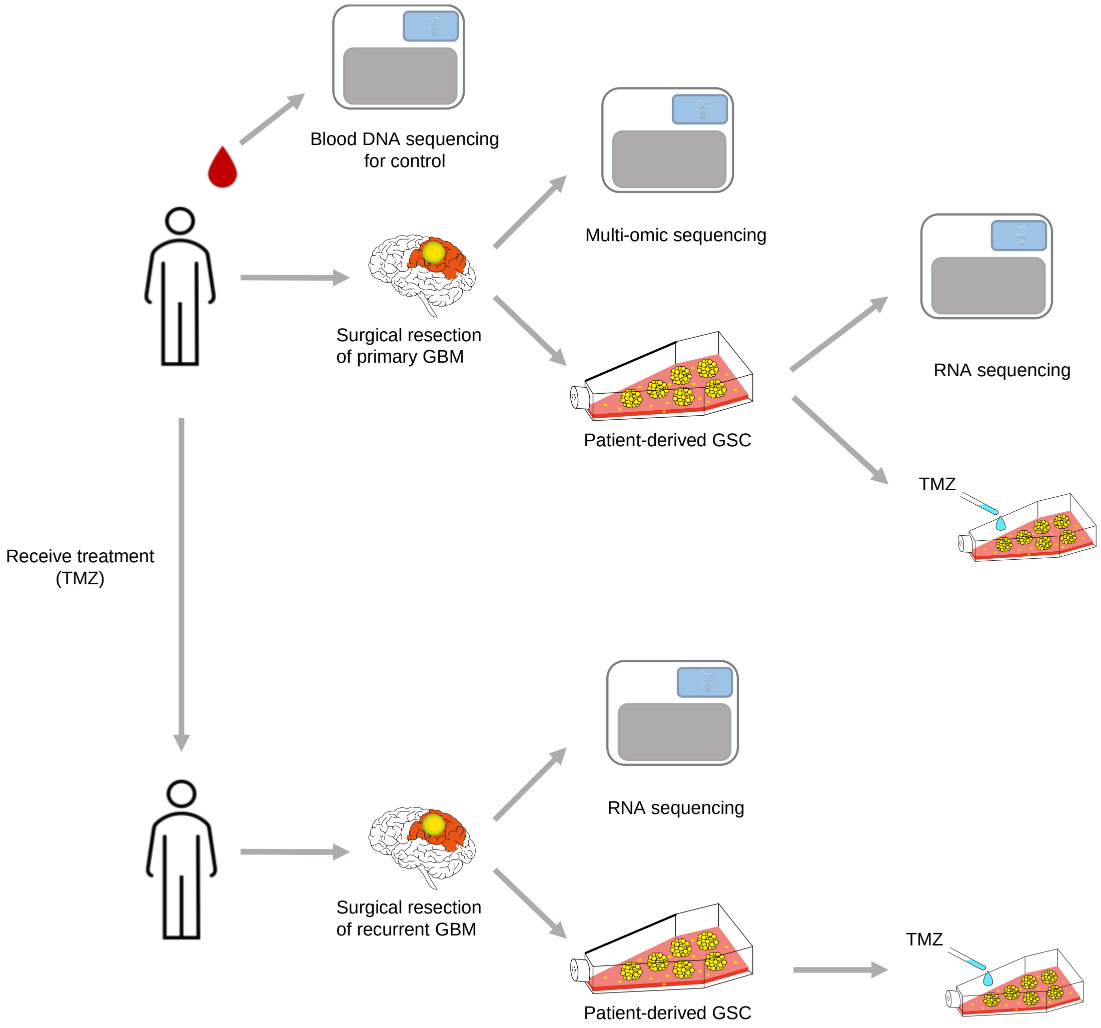

Fig. S1. **Timeline of sample acquisition, sequencing, in vitro culture and TMZ screening.**

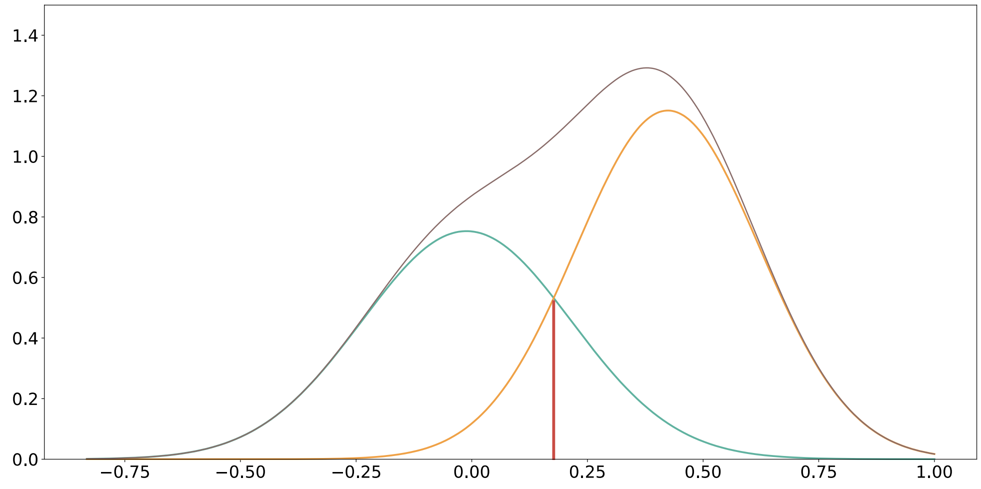

Fig. S2. Gaussian Mixture Model used to identify genes with the same expression profile between patient-derived cells (PDCs) and tumor tissues. Spearman's rank correlation coefficients of all genes (n = 20,956) between matched GSCs and tumor tissues of 12 patients from the main cohort is shown on the x-axis. Normalized probability density is denoted on the y-axis. The two weighted gaussian components are shown in green and orange curves. The intersection of the two gaussians is shown (red line) to denote the cutoff (> 0.177) of Spearman's rank correlation coefficients with higher probability in the gaussian component of positive correlation (orange curve). The overall distribution is shown as a black curve.

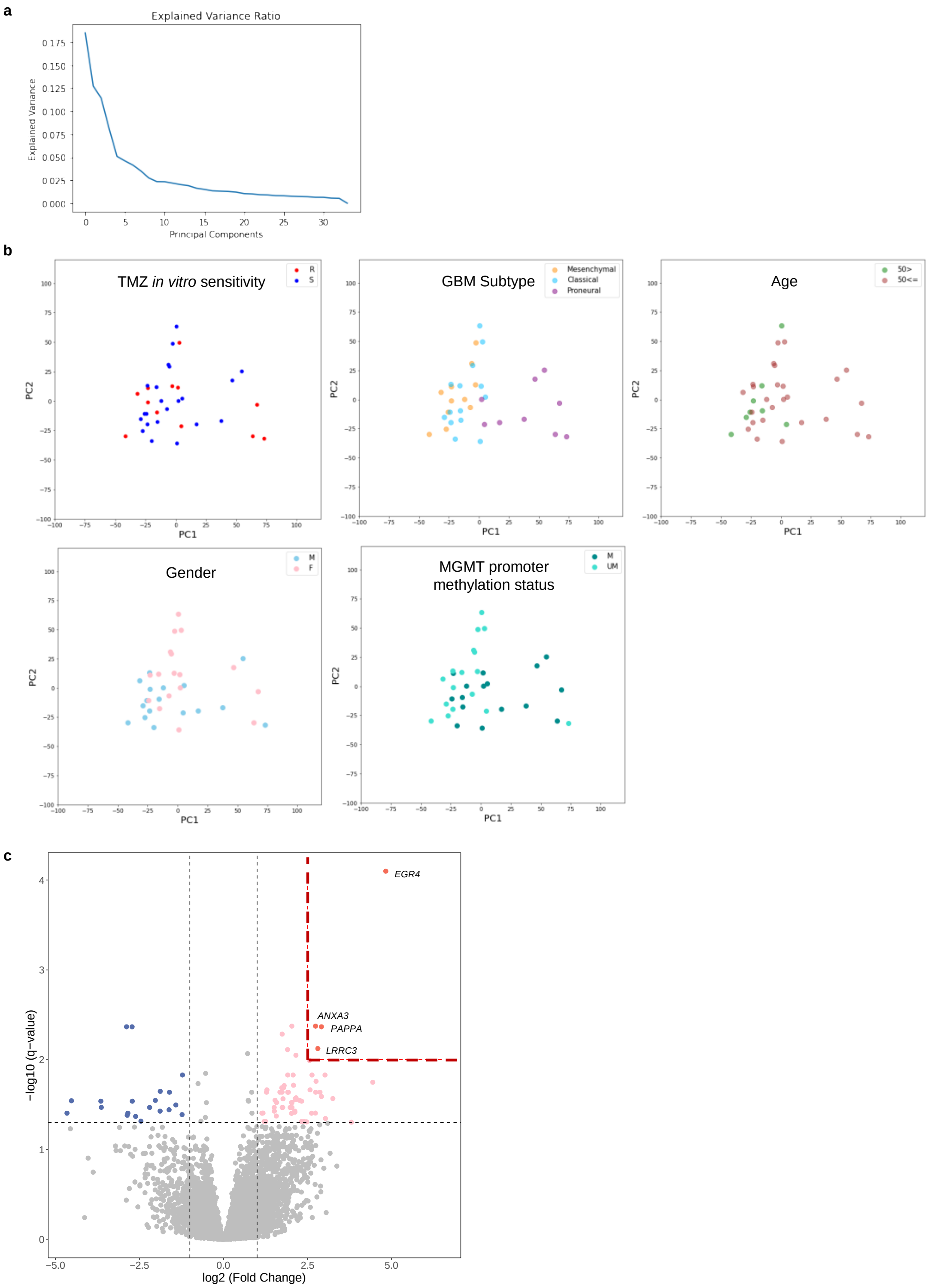

Fig. S3. Principal Component Analysis and differentially expressed gene analysis on 34 tissue RNA-seq samples. a Principal components and explained variance on 34 RNA-seq samples from the main cohort. Conserved genes were used as feature input. **b** PCA plots with principal component 1 (PC1) and principal component 2 (PC2) on 34 tissue RNA-seq samples colored by annotated categories. R, TMZ-resistant; S, TMZ-sensitive; M, male; F, female; M, methylated; UM, unmethylated. **c** Volcano plot showing the differentially expressed genes from DESeq2 analysis. Black dashed lines are less stringent cut-offs (|log2 fold change| > 1, adjusted P < 0.05) to show the distribution of other potential DEGs (pink, TMZ-resistant up-regulated genes; blue, TMZ-sensitive up-regulated genes). Red dashed lines are the stringent cut-off we used (log2 fold change > 2.5, adjusted P < 0.01) to define 4 TMZ-resistant marker genes (red).

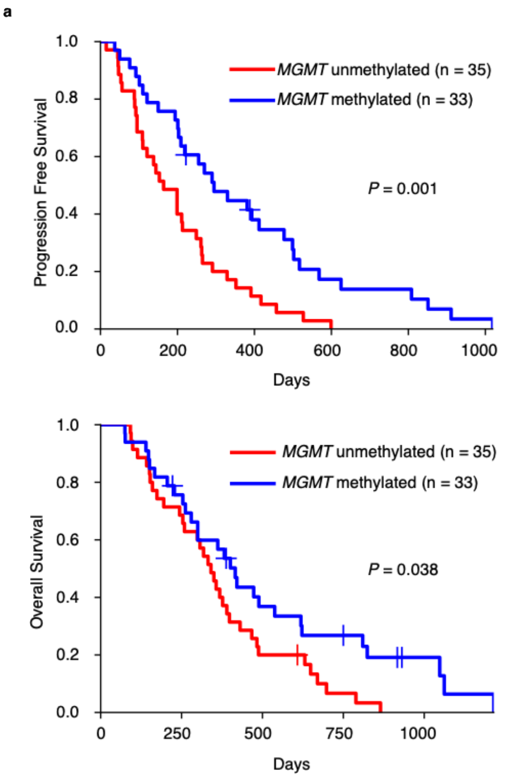

b

Fisher exact *P* = 0.0156		*In vitro* TMZ Screening	
		Resistant	Sensitive
MGMT promoter status	Unmethylated	20	15
	Methylated	9	24

Fig. S4. Association of MGMT promoter methylation status to survival and *in vitro* TMZ screening in the main cohort. a Progression free survival (upper panel) and overall survival (bottom panel) of the *MGMT* unmethylated (red) and *MGMT* methylated (blue) patients in the main cohort (n = 68)**. b** Fisher's exact test contingency table for *In vitro* TMZ sensitivity and *MGMT* promoter status association in the main cohort.

CDK4 (0.5, 1.58, 1.0)		WES norm	WES gain	WES amp
GS	norm	12	0	0
	gain	0	1	0
	amp	0	0	4

MDM2 (0.5, 1.58, 0.66)		WES norm	WES gain	WES amp
GS	norm	14	1	0
	gain	0	0	0
	amp	0	0	2

CDKN2A (-0.5, -1.58, 0.90)		WES norm	WES loss	WES del
GS	norm	3	1	0
	loss	0	3	0
	del	0	0	10

PDGFRA (0.5, 1.58, 0.67)		WES norm	WES gain	WES amp
GS	norm	15	0	0
	gain	0	0	0
	amp	0	0	2

EGFR (0.3, 1.58, 0.78)		WES norm	WES gain	WES amp
GS	norm	3	3	0
	gain	0	3	0
	amp	0	0	8

PTEN (-0.5, -1.58, 0.53)		WES norm	WES loss	WES del
GS	norm	6	2	0
	loss	1	7	1
	del	0	0	0

All Genes (avg. F- score = 0.90)		WES norm	WES loss or gain	WES del or amp
GS	norm	53	6	0
	loss or gain	1	14	1
	del or amp	0	0	26

Fig. S5. Copy number estimation by GliomaSCAN. The tables show number of copy number altered samples estimated by WES and GliomaSCAN. A total of 17 samples that had both WES and GliomaSCAN data was used in the tables. Three numbers presented in the parenthesis indicate the cut-offs for copy number score from GliomaSCAN to distinguish normal from gain/loss (cut-off-1) and gain/loss from amplification/deletion (cut-off-2). The last number shown is the average F-score calculated by cut-off-1 and cut-off-2. Note that for copy number loss or deleted genes, -0.5 and -1.58 log2 ratio of tumor and normal were used as cut-off-1 and cut-off-2 for both WES and GliomaSCAN data, and 0.5 and 1.58 were used as cut-off-1 and cut-off-2 for copy number gain or amplified genes. But in the case of EGFR, GliomaSCAN's copy number result was less accurate and therefore 0.3 and 1.58 were used as cut-offs to increase compatibility with WES results. The cut-offs were used to determine the copy number alteration status of those without WES. The last table shows combined prediction for all six genes.

CDK4 (0.62, 0.92, 0.82)		WES norm	WES gain	WES amp
RNA	norm	31	2	0
	gain	0	1	0
	amp	0	0	4

MDM2 (0.75, 0.9, 1.0)		WES norm	WES gain	WES amp
RNA	norm	34	0	0
	gain	0	2	0
	amp	0	0	2

CDKN2A (0.17, 0.76, 0.60)		WES norm	WES loss	WES del
RNA	norm	4	0	0
	loss	4	6	7
	del	2	1	14

PDGFRA (0.79, 0.79, 0.66)		WES norm	WES gain	WES amp
RNA	norm	35	0	0
	gain	0	0	0
	amp	0	0	3

EGFR (0.58, 0.98, 0.74)		WES norm	WES gain	WES amp
RNA	norm	7	2	0
	gain	2	9	2
	amp	0	4	12

PTEN (0.98, 1.0, 0.57)		WES norm	WES loss	WES del
RNA	norm	14	1	3
	loss	2	17	1
	del	0	0	0

All Genes (avg. F- score = 0.82)		WES norm	WES loss or gain	WES del or amp
RNA	norm	125	5	3
	loss or gain	8	35	10
	del or amp	0	5	35

Fig. S6. Copy number estimation by RNA-seq. The tables show number of copy number altered samples estimated by WES and RNA-seq. Total of 38 samples with both WES and RNA-seq data was used. Three numbers presented in the parenthesis indicate the cut-offs for copy number score from RNA-seq to distinguish normal from gain/loss (cut-off-1) and gain/loss from amplification/deletion (cut-off-2). The last number shown is the average F-score calculated by cut-off-1 and cut-off-2. These cut-offs were used to determine the copy number alteration status of those without WES. The last table shows combined prediction for all six genes.

a

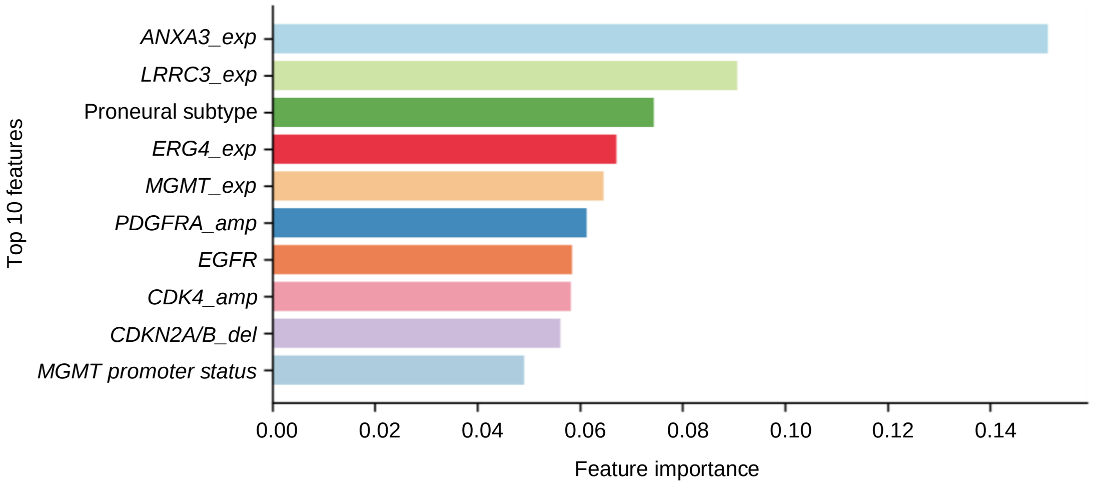

b

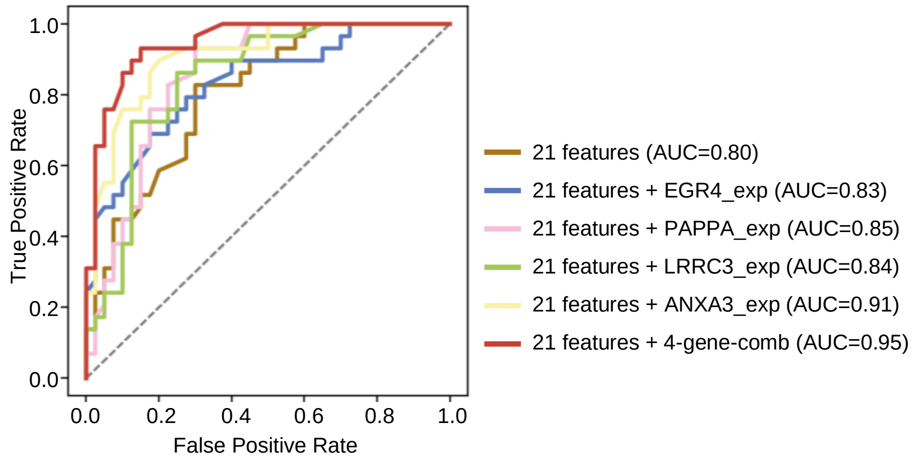

Fig. S7. Machine learning model feature importance. a Top 10 feature importance in the model. **b** ROC curves showing the combinatorial contribution of 4 TMZ-Resistant markers. 21 features without adding 4 TMZ-Resistant expression markers (*EGR4, PAPPA, LRRC3, ANXA3*, shown in **Fig. 4a**). 4-gene-comb denotes the combination of the 4 expression markers.

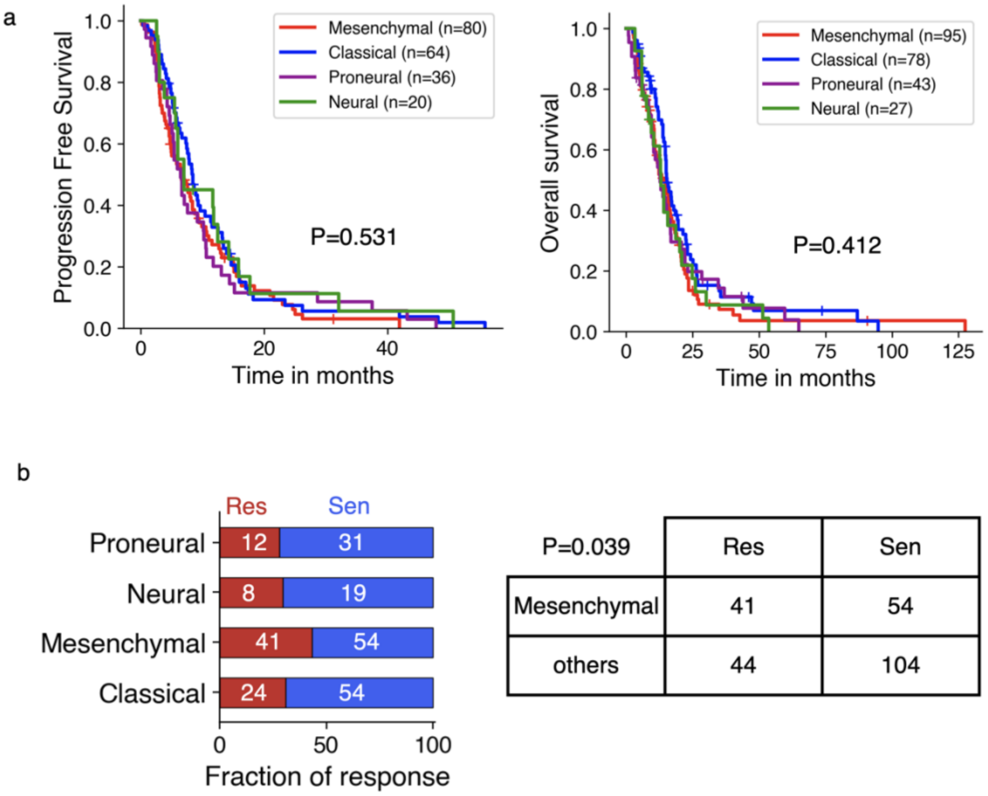

Fig. S8. Correlations between GBM subtypes and TMZ response. a Survival curves of TCGA IDH-wt, TMZ treated primary GBM samples separated by four GBM subtypes. P-value was computed via multivariate log-rank test. The four GBM subtypes are from Verhaak *et al.*[20]. **b** Distribution of the GBM subtypes in the TCGA cohort by TMZ response predicted from the machine learning model.

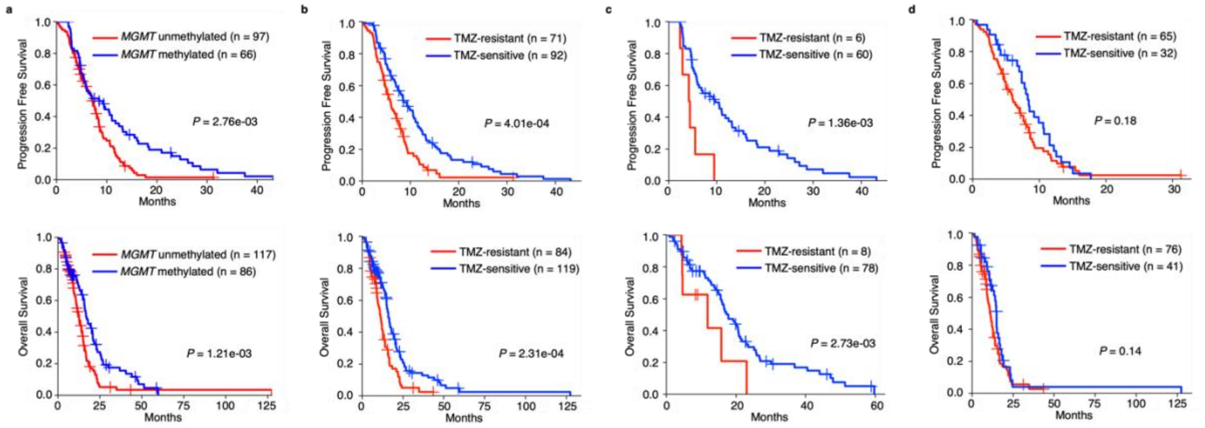

Fig. S9. Comparison of survival prediction in TCGA cohort. Kaplan-Meier survival curves of 203 MGMT status available, IDH-wt, TMZ treated, primary GBM samples from TCGA grouped by **a** MGMT promoter methylation status and **b** machine learning model. **c** Kaplan-Meier survival curves of MGMT methylated TCGA samples grouped by machine learning model. **d** Kaplan-Meier survival curves of MGMT unmethylated TCGA samples grouped by machine learning model.

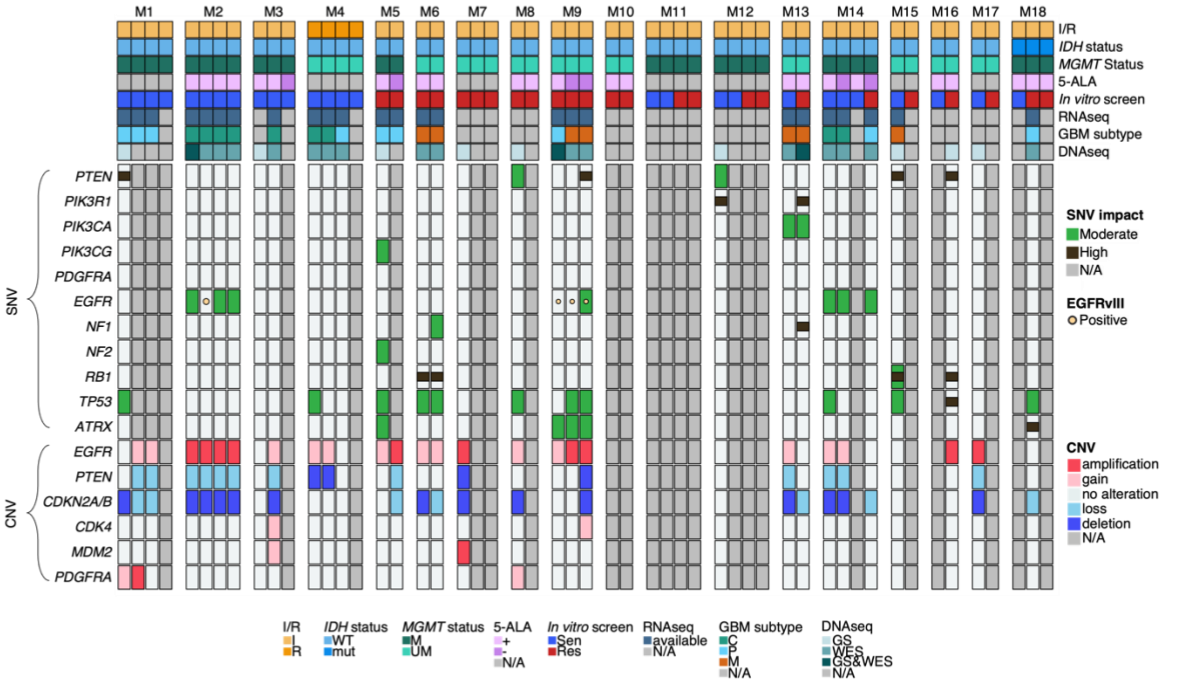

Fig. S10. Genomic landscape of multi-sector samples. I, initial tumor; R, recurrent tumor; WT, wild-type; mut, mutant; M, methylated; UM, unmethylated; N/A, not available; Sen, TMZ-sensitive; Res, TMZ-resistant; C, classical; P, proneural; M, mesenchymal; GS, GliomaSCAN; Moderate: Missense or Inframe deletion; High: Frameshift, Stop gained, Splice donor or Splice acceptor.

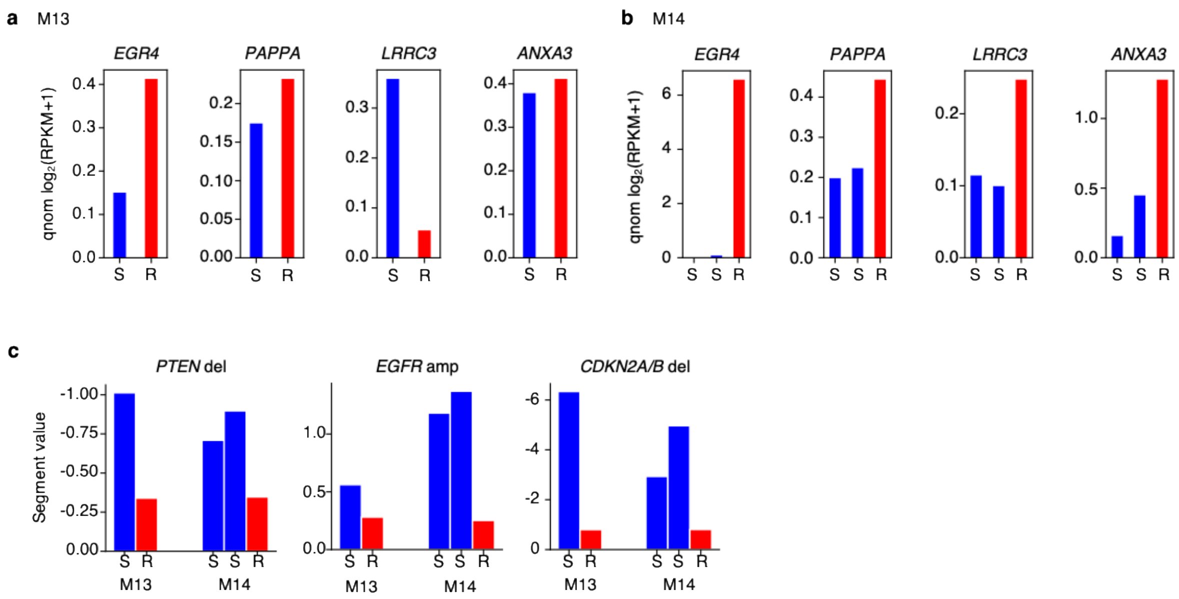

Fig. S11. TMZ-resistant marker expression and CNV comparison in patient M13 and M14. a,b Gene expression comparison of TMZ-Resistant marker genes in multi-sector samples of **(a)** patient M13 and **(b)** patient M14. **c** Segment value comparison of TMZ-sensitive and TMZ-resistant samples within patient. Blue, expression level in TMZ-Sensitive samples; red, expression level in TMZ-Resistant samples. S, TMZ-sensitive; R, TMZ resistant.

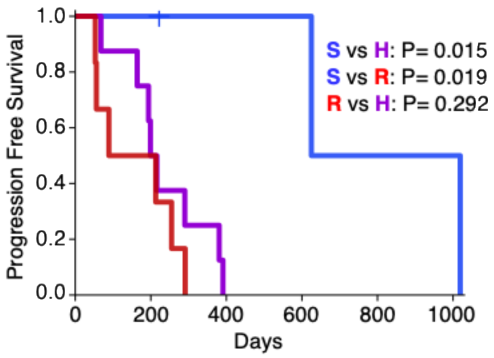

Fig. S12. Progression free survival difference in patients with multi-sector samples. S, all sensitive (M1~M3); H, heterogeneous (M11~M18); R, all resistant (M5~M10). P-values calculated by logrank test.
