# Supplementary material for: Pharmacogenomic profiling reveals molecular features of chemotherapy resistance in IDH wild-type primary glioblastoma
Source: Genome Med. 2023 Mar 13;15:16. doi: 10.1186/s13073-023-01165-8 (PMC10010007; doi:10.1186/s13073-023-01165-8)
Supplement: Supplementary file 3 — Additional file 3. Supplementary methods. [file 13073_2023_1165_MOESM3_ESM.pdf]

Supplementary Methods

Prediction of CNA status from RNAseq data---1
Supplementary references---3

Prediction of CNA status from RNAseq data

A logistics regression model was used to predict the CNA status as

$$\Pr(\mathrm{x_i}) = \frac{e^{\sum_{k=1}^{T} f_k(x_i)}}{1 + e^{\sum_{k=1}^{T} f_k(x_i)}}, f_k \in F$$

$$\hat{y_i} = \begin{cases} 1, \Pr(\mathrm{x_i}) > 0.5 \\ 0, \Pr(\mathrm{x_i}) \leq 0.5 \end{cases}$$

The x_i is the vector of gene expression of sample i, containing the RPKM value x_{ij} of gene j in sample i. We first used the pan-glioma dataset to train our model[1], and the CNA status of sample i was defined as $y_i \in (0, 1)$, where 0 represents normal and 1 represents amplification or homo-deletion according to interest. To reduce the dimension of the data, a set S was defined as genes related to the target gene, which is: (1) on the same chromosome with the target gene, (2) in the same pathway recorded in KEGG database[2], (3) protein-protein interaction with target gene recorded in STRING database[3]. Only genes in S is used in the model ($j \in S$). $f_k(x_i)$ is the base classifier, and T is the number of base classifiers, which was set to 100 according to experience.

Decision tree was employed to be the base classifier and the tree boosting was achieved by XGBoost[4], which generate trees by optimizing the objective function obj:

$$obj = \sum_{i=1}^{n} L(y_i, \hat{y_i}) + \sum_{k=1}^{T} \Omega(f_k)$$

In this equation, the loss function $L(y_i, \hat{y_i})$, which is used to improve the accuracy, is:

$$L(\mathrm{y_i}, \hat{y_i}) = I_i, \qquad I_i = \begin{cases} 1, y_i \neq \hat{y_i} \\ 0, y_i = \hat{y_i} \end{cases}$$

And the regularization function $\Omega(f_k)$, which is used to reduce the complexity of the model, is:

$$\Omega(f_k) = \gamma L + \frac{1}{2}\lambda \sum_{l=1}^{L} w_l^2$$

Here w represents scores on decision tree's leaves, and L is the number of decision tree's leaves. For two learning parameters λ and γ, default values were used in our model.

We next applied the model to our dataset for the calculation of $\hat{c_i} = \mathrm{C}(\hat{y_i})$ as the following formula shows. To enhance the accuracy of the prediction and further distinguish the level of copy number alteration (i.e., gain and amplification, loss and deletion), we utilized 38 samples with WES and

matched RNA-seq. We calculated two new cut-offs (a_j and b_j) for each gene *j* by selecting the cut-offs with optimal average F-score from the precision and recall of the test (Fig. S6).

$$\widehat{c_i} = \begin{cases} 3, \widehat{y_i} > b_j \\ 2, a_j \leq \widehat{y_i} \leq b_j \\ 1, \widehat{y_i} < a_j \end{cases}$$

Final copy number status was defined as $c_i \in (1, 2, 3)$, where 1 represents normal, 2 represents gain or loss, and 3 is for amplification or homo-deletion according to interest.

Supplementary references

1. Ceccarelli M, Barthel FP, Malta TM, et al. Molecular Profiling Reveals Biologically Discrete Subsets and Pathways of Progression in Diffuse Glioma. *Cell*. 2016;164(3):550-563. doi:10.1016/j.cell.2015.12.028
2. Kanehisa M, Goto S. KEGG: Kyoto Encyclopedia of Genes and Genomes. *Nucleic Acids Res*. 2000. doi:10.1093/nar/28.1.27
3. Szklarczyk D, Franceschini A, Wyder S, et al. STRING v10: Protein-protein interaction networks, integrated over the tree of life. *Nucleic Acids Res*. 2015. doi:10.1093/nar/gku1003
4. Chen T, Guestrin C. XGBoost: A scalable tree boosting system. In: *Proceedings of the ACM SIGKDD International Conference on Knowledge Discovery and Data Mining*. ; 2016. doi:10.1145/2939672.2939785
